# A Mechanism for Priming and Realignment during Influenza A Virus Replication

**DOI:** 10.1128/JVI.01773-17

**Published:** 2018-01-17

**Authors:** Judith Oymans, Aartjan J. W. te Velthuis

**Affiliations:** a Sir William Dunn School of Pathology, University of Oxford, Oxford, United Kingdom; b University of Cambridge, Department of Pathology, Division of Virology, Addenbrooke's Hospital, Cambridge, United Kingdom; St. Jude Children's Research Hospital

**Keywords:** influenza A virus, RNA-dependent RNA polymerase, viral replication, priming loop, realignment, ApG

## Abstract

The influenza A virus genome consists of eight segments of single-stranded RNA. These segments are replicated and transcribed by a viral RNA-dependent RNA polymerase (RdRp) that is made up of the influenza virus proteins PB1, PB2, and PA. To copy the viral RNA (vRNA) genome segments and the cRNA segments, the replicative intermediate of viral replication, the RdRp must use two promoters and two different *de novo* initiation mechanisms. On the vRNA promoter, the RdRp initiates on the 3′ terminus, while on the cRNA promoter, the RdRp initiates internally and subsequently realigns the nascent vRNA product to ensure that the template is copied in full. In particular, the latter process, which is also used by other RNA viruses, is not understood. Here we provide mechanistic insight into priming and realignment during influenza virus replication and show that it is controlled by the priming loop and a helix-loop-helix motif of the PB1 subunit of the RdRp. Overall, these observations advance our understanding of how the influenza A virus initiates viral replication and amplifies the genome correctly.

**IMPORTANCE** Influenza A viruses cause severe disease in humans and are considered a major threat to our economy and health. The viruses replicate and transcribe their genome by using an enzyme called the RNA polymerases. To ensure that the genome is amplified faithfully and that abundant viral mRNAs are made for viral protein synthesis, the RNA polymerase must work correctly. In this report, we provide insight into the mechanism that the RNA polymerase employs to ensure that the viral genome is copied correctly.

## INTRODUCTION

Influenza A viruses (IAVs) are important pathogens that cause seasonal epidemics and occasional pandemics in humans. The IAV genome comprises eight segments of single-stranded, negative-sense viral RNA (vRNA) that exist in the context of viral ribonucleoproteins (vRNPs) (1). These vRNPs consist of one vRNA segment, a copy of the viral RNA-dependent RNA polymerase (RdRp), and a helical coil of the viral nucleoprotein (NP). During IAV infections, the vRNPs are released from viral particles and imported into the nucleus of the host cell. In the nucleus, the vRNPs replicate the vRNAs via a cRNA intermediate, and they transcribe the vRNAs to form viral mRNAs (1, 2). The latter molecules are exported from the nucleus and translated by cellular ribosomes, while cRNAs are bound by new NP and RdRp molecules in order to form cRNPs capable of synthesizing new vRNAs (3).

**FIG 1:**
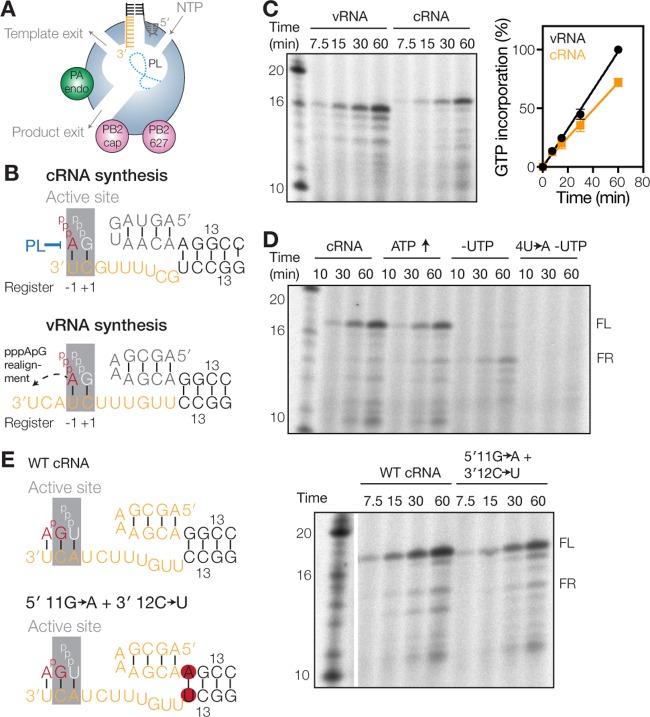
Failed priming and realignment events can be detected *in vitro*. (A) Model of the influenza A virus RdRp. The core of the RdRp is shaded blue, the PA endonuclease (PA endo) is in green, and the PB2 cap-binding domain (PB2 cap) and 627 domain (PB2 627) are in pink. The priming loop (PL) is indicated as a dotted line. (B) Schematics of IAV initiation during cRNA and vRNA synthesis. The priming loop is indicated in blue. Active-site (gray) positions −1 and +1 are indicated below each schematic. (C) Time course of ApG extension on a vRNA or cRNA promoter. The graph shows the percentage of [α-^32^P]GTP incorporation relative to activity on the vRNA promoter. Error bars indicate standard deviations (*n* = 3). (D) Time course of ApG extension on a wild-type or 4U→A mutant cRNA promoter. The addition of 0.5 mM ATP or the omission of UTP is indicated. (E) Schematic of ApG extension on a wild-type (WT) cRNA promoter or a promoter where the first G-C base pair of the promoter duplex was mutated to A-U (5′ 11G→A+3′ 12C→U). The gel image shows results of an ApG extension assay on the wild-type cRNA promoter or the 5′ 11G→A+3′ 12C→U promoter mutant.

Both IAV replication and transcription are catalyzed by the RdRp (1, 2). This RdRp is a 250-kDa heterotrimer that consists of the viral proteins polymerase basic protein 1 (PB1), PB2, and polymerase acidic protein (PA) (1, 4). The PB1 subunit, the N-terminal one-third of PB2, and the C-terminal two-thirds of PA form the conserved RdRp domain (5–7), while the remaining parts of PA and PB2 form flexible domains at the periphery of the polymerase core (Fig. 1A). These domains are important for cleaving cellular host mRNAs, a process that yields capped RNA primers that are essential for viral transcription (1, 4).

The viral RdRp binds the 5′- and 3′-terminal ends of the vRNA or cRNA, also called the vRNA or cRNA promoter, respectively (8–10), via a binding pocket above the nucleoside triphosphate (NTP) channel (5, 6, 11). The 3′ end of either promoter can translocate to the active site (12) (Fig. 1A), allowing *de novo* initiation on the terminal 3′ UC of the vRNA (Fig. 1B) or *de novo* initiation at positions 4U and 5C of the 3′ terminus of the cRNA (Fig. 1B) (13, 14). However, the two *de novo* initiation mechanisms are markedly different. Terminal *de novo* initiation on a vRNA promoter, but not internal *de novo* initiation on a cRNA promoter, critically depends on the PB1 priming loop (Fig. 1B) (13). In contrast, internal initiation requires a realignment of the pppApG initiation product to the 3′ 1U and 2C bases of the cRNA prior to elongation (13, 14) (Fig. 1B). A failure to perform this realignment step would generate a vRNA lacking the first 3 nucleotides (nt) of the canonical vRNA 5′ terminus. Since the vRNA 5′ terminus is part of the vRNA promoter and is critical for the activity and conformational changes of the influenza virus RdRp (5–7), such a truncated vRNA would likely not support efficient cRNA and mRNA synthesis. Current evidence suggests that a number of negative-strand RNA viruses use a similar or related realignment process to ensure the faithful replication of their genomes (14–16), but the molecular mechanism that controls these realignment processes is currently not understood.

In this study, we investigated the priming and realignment mechanism using a combination of cell-based RNP reconstitution assays, structure-guided mutagenesis, and *in vitro* activity assays. We show that the IAV RdRp uses its priming loop to enforce priming and realignment and, thus, correct vRNA synthesis during replication. Our observations provide novel mechanistic insight into IAV RNA synthesis and expand our current view of the role of the priming loop.

(This article was submitted to an online preprint archive [17].)

## RESULTS

### Priming and realignment are essential for viral RNA synthesis.

The IAV RdRp catalyzes mRNA and cRNA synthesis from the vRNA promoter and vRNA synthesis from the cRNA promoter. We previously showed that the initiation activities of the IAV RdRp on these two promoters are similar during viral replication (13). However, a side-by-side comparison of the extension activity of the RdRp on these two promoters shows that the RdRp activity is ∼30% weaker on the cRNA promoter than on the vRNA promoter (Fig. 1C) (note that the full-length [FL] products of the vRNA and cRNA promoters, 14-nt and 15-nt products, respectively, migrate near the 16-nt and 17-nt marker bands, because the ApG that primes the reaction lacks a 5′ phosphate, which reduces the negative charge and migration of the viral products relative to the 5′-phosphorylated marker [13]). A similar observation was recently made for the activities of influenza B virus RdRp on the influenza B virus vRNA and cRNA promoters (18). This difference in promoter activity may be explained, at least in part, by the priming and realignment mechanism that the RdRp uses during initiation on the cRNA promoter (Fig. 1B) or the difference in the affinities of the RNA polymerase for the two templates (12). So far, the efficiency of the former and the mechanism behind the realignment process have not been studied in detail.

To study how the IAV RdRp coordinates priming and realignment, we attempted to measure the formation of failed realignment (FR) products in ApG extension assays using influenza A/WSN/33 (H1N1) virus RdRp preparations. We decided to use the fact that ApG extension after a realignment step requires UTP incorporation, while a failed realignment event utilizes ATP for extension (Fig. 1B). To favor the latter event, we either doubled the ATP concentration or omitted UTP from the reaction mixture. Only under UTP-free conditions did we observe a loss of the 15-nt FL product and the appearance of a 12-nt main product (Fig. 1D). Moreover, this 12-nt product was not synthesized in reactions where 4U of the 3′ cRNA strand was mutated to A (4U→A) to prevent ApG priming at position 4/5 and reduce internal elongation (Fig. 1D), confirming that the FR product was dependent on internal ApG extension.

To investigate whether the amount of the FR product was dependent on the stability of the model promoter duplex, we replaced the first G-C base pair of the duplex of the cRNA promoter with an A-U base pair to make the duplex less stable (Fig. 1E). No increase in FR product formation was observed in reaction mixtures containing the mutant cRNA promoter duplex (Fig. 1E), suggesting that the duplex of the model cRNA promoter does not influence the priming and realignment mechanism. Together, these observations thus suggest that failed priming and realignment events can be detected and identified *in vitro* but that they do not occur frequently. This implies that the efficiency of priming and realignment is relatively high and that the difference in promoter activity (Fig. 1C) may be largely explained by the different efficiencies of binding of the RdRp to the two viral promoters (12).

### PB1 V273 modulates priming and realignment during vRNA synthesis.

**FIG 2:**
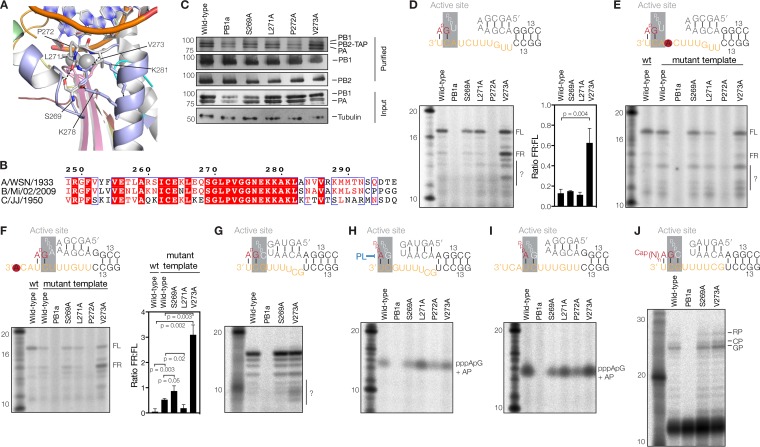
PB1 V273 affects priming and realignment. (A) Superposed structure of the bat influenza A virus RdRp (PDB accession number 4WSB) with the poliovirus 3D^pol^ RdRp elongation complex (PDB accession number 3OL7). For the 3D^pol^ complex, the template strand, nascent strand, and magnesium ions are shown. For the bat influenza A virus RdRp, the PB1 subunit is shown in light blue, with polymerase motifs A, C, D, and F shown in yellow, pink, red, and pale green, respectively. Polar interactions between amino acids of the helix-loop-helix structure are indicated with dotted lines. Additional side chains are shown for reference. (B) Amino acid alignment of the PB1 helix-turn-helix structure of the palm subdomain. PB1 sequences of influenza A/WSN/33 (H1N1), influenza B/Michigan/22687/09, and influenza C/JJ/50 viruses are shown. Identical residues are shaded red, and conserved residues are surrounded with blue boxes. Secondary-structure annotations are based on data reported under PDB accession number 4WSB. (C) SDS-PAGE and silver staining of recombinant influenza A virus RdRps isolated from HEK 293T cells or Western blot analysis of PB1 and PB2 in isolated RdRps. The bottom panel shows the expression of PB1 and PA RdRp subunits in HEK 293T cells. A tubulin loading control is also shown. (D) ApG extension on a cRNA promoter. The graph shows the ratios of the quantified FR and FL signals of three independently purified RdRp sets. The *P* value was determined by using an unpaired *t* test. Unknown products are indicated with a question mark. (E) ApG extension on the 4U→A mutant cRNA promoter. The question mark indicates an increase in the amount of unknown RNA products. (F) ApG extension on the 1U→A mutant cRNA promoter. The graph shows the mean FR-to-FL product ratios for three independently purified RdRp sets. The *P* values were determined by using an unpaired *t* test. (G) ApG extension on a vRNA promoter. Unknown products are indicated with a question mark. In each graph, the error bars indicate standard deviations (*n* = 3). (H) Terminal pppApG synthesis on a cRNA promoter in the presence of ATP and [α-^32^P]GTP. The reaction mixtures were treated with alkaline phosphatase (AP) to better separate the radioactive product from the nonincorporated [α-^32^P]GTP and free phosphates. (I) Internal pppApG synthesis on a vRNA promoter. (J) Extension of a radiolabeled capped 11-nucleotide-long RNA primer ending in 3′ AG. This extension reaction yields a product initiated at G3 (GP) or a product initiated at 2C (CP) of the vRNA promoter and an additional realignment product (RP) (40).

Realignment of the pppApG initiation product during vRNA synthesis is likely controlled by the RdRp structure. Inside the RdRp, the nascent A-form duplex, consisting of template and product RNAs, is guided away from the active site by a conserved helix-turn-helix structure (Fig. 2A). To investigate whether residues in this structure contributed to the priming and realignment mechanism, we engineered an alanine substitution of conserved PB1 residue S269, L271, P272, or V273 at the top of the helix-turn-helix (Fig. 2B), creating S269A, L271A, P272A, and V273A mutants, respectively. A PB1 mutant containing alanine substitutions of two critical active-site aspartates (PB1 DD445–446AA [PB1a]) (19) was used as a negative control. Using IgG-Sepharose purification followed by SDS-PAGE analysis and silver staining or Western blotting, we verified that the mutations had no effect on heterotrimer formation (Fig. 2C).

To investigate the effect of the mutations on realignment efficiency, we performed ApG extension assays on a cRNA promoter and analyzed the reactions by 20% denaturing PAGE. No change in FR product formation was observed in reaction mixtures containing the PB1 S269A and L271A mutants (Fig. 2D). In contrast, the P272A mutant failed to extend the ApG dinucleotide (Fig. 2D), while the V273A produced significantly more FR than did the wild type (Fig. 2D). In addition, V273A mutant produced RNAs that migrated faster than the FR product, but the nature of these RNA species is presently unknown. To confirm that the V273A FR product had been produced through internal elongation, we measured the activity of the V273A mutant on the 4U→A mutant promoter to reduce internal ApG binding and observed a substantial reduction in the FR signal without an impairment of the production of the FL product (Fig. 2E). In support of this observation, mutation of 1U of the cRNA 3′ strand to A (1U→A), which reduces ApG priming at position 1U/2C, reduced FL product formation for all RdRps tested and increased the FR/FL product ratio ∼3-fold for the V273A mutant (Fig. 2F). To verify that the V273A mutation specifically affected the priming and realignment mechanisms and that no other RdRp activities were affected, we performed ApG extensions on the vRNA promoter (Fig. 2G) and *de novo* initiation and transcription initiation assays (Fig. 2H to J). We found no effect of the V273A mutation on polymerase activity in any of these assays. In contrast, mutations S269A, L271A, and P272A had small effects on *de novo* initiation (Fig. 2H to J). Overall, these observations suggest that V273 at the tip of the PB1 helix-turn-helix primarily affects the priming and realignment process.

**FIG 3:**
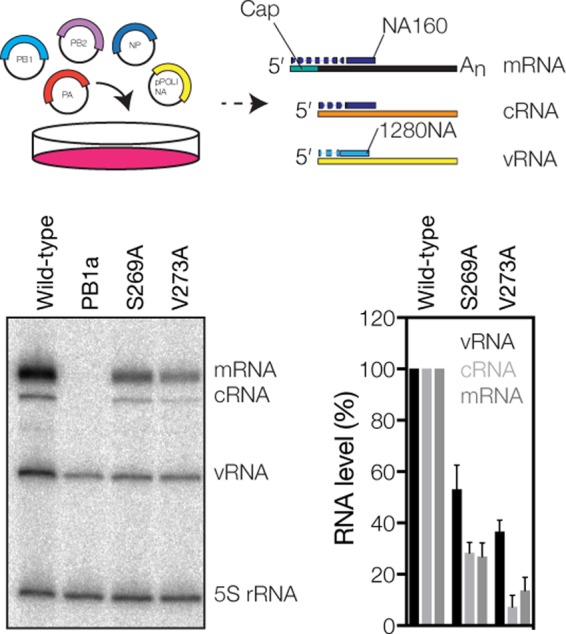
PB1 V273 affects RNA synthesis in cell culture. Schematic of the RNP reconstitution assay and analysis of the steady-state segment 6 RNA levels. The graph shows the mean RNA levels from three independent experiments after subtraction of the PB1 active-site control signal. Error bars indicate standard deviations (*n* = 3).

We next investigated the activity of V273A in cell culture. To this end, we used a minireplicon assay that relies on the reconstitution of vRNPs from plasmid-expressed IAV RdRp subunits, NP, and a segment 6 (neuraminidase [NA]-encoding) vRNA template (Fig. 3). After RNA extraction, the synthesis of the viral RNA species (vRNA, cRNA, and mRNA) was measured by using primer extensions of 5′-radiolabeled primers (Table 1). As shown in Fig. 3, viral RNA synthesis by the V273A mutant, but also by the S269A mutant, was impaired compared to the wild type and was found to have differential effects on cRNA and mRNA synthesis. These observations corroborate previously reported RNP reconstitutions with the same mutants (20) and experiments showing that a V273A, V273L, or V273D mutation reduces the fitness of A/WSN/33 (H1N1) viruses by >90% (21). Based on these observations, we suggest that an inefficient realignment of the nascent vRNA results in the synthesis of truncated vRNA products that may not be bound by the IAV RdRp. In turn, these truncated vRNAs may be degraded by the host cell, which restricts viral cRNA and mRNA synthesis to primary RNA synthesis and reduces viral RNA levels without directly affecting the mechanism of transcription or cRNA synthesis. Overall, these results imply that correct realignment and/or internal initiation is essential for influenza virus replication and transcription and that this process is modulated, at least in part, by PB1 residue V273.

**TABLE 1:** Primers used for PB1 mutagenesis and primer extension

Mutant	Primer direction^*a*^	Sequence (5′–3′)
PB1 Δ648–651	Fw	GAACAATGCAGTGATAATGCCAGGCGCCAAAAACATGGAGTATGATGC
	Rv	GCATCATACTCCATGTTTTTGGCGCCTGGCATTATCACTGCATTGTTC
PB1 Δ631–662	Fw	GCAACCCACTGAACCCAACACACTCCTGGATCCCC
	Rv	GGGGATCCAGGAGTGTGTTGGGTTCAGTGGGTTGC
PB1 Δ642–656	Fw	AACCATAAAGACATTGAATCAGTGAACTATGATGCTGTTGCAACAACA
	Rv	TGTTGTTGCAACAGCATCATAGTTCACTGATTCAATGTCTTTATGGTT
PB1 Δ656–662	Fw	ATGGTCCAGCCAAAAACATGACACACTCCTGGATCCCC
	Rv	GGGGATCCAGGAGTGTGTCATGTTTTTGGCTGGACCAT
PB1 Δ631–642	Fw	GCAACCCACTGAACCCAGCAGTGATAATGCCAGCAC
	Rv	GTGCTGGCATTATCACTGCTGGGTTCAGTGGGTTGC
PB1 Δ636–642	Fw	CTGAACCCATTTGTCAACCATAAAGCAGTGATAATGCCAGCAC
	Rv	GTGCTGGCATTATCACTGCTTTATGGTTGACAAATGGGTTCAG
PB1 Δ631–635	Fw	GCAACCCACTGAACCCAGACATTGAATCAGTGAACAATGCA
	Rv	TGCATTGTTCACTGATTCAATGTCTGGGTTCAGTGGGTTGC
1280 NA (vRNA)	Fw	TGGACTAGTGGGAGCATCAT
NA160 (mRNA/cRNA)	Rv	TCCAGTATGGTTTTGATTTCC
5S100	Rv	TCCCAGGCGGTCTCCCATCC

a Fw, forward; Rv, reverse.

### The priming loop is important for viral RNA synthesis in cell culture.

**FIG 4:**
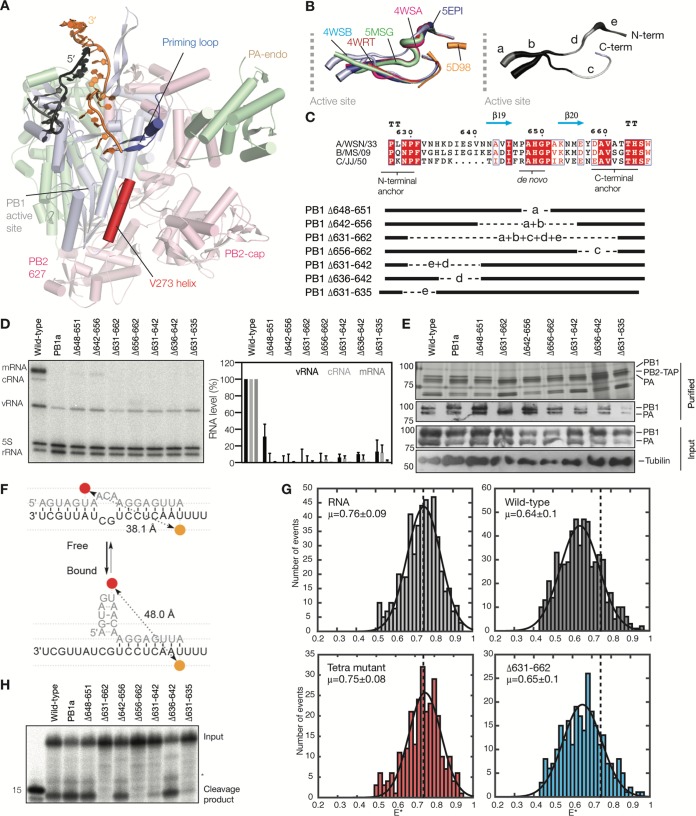
Deletions in the priming loop affect viral RNA synthesis in cell culture. (A) Position of the priming loop relative to the active site, the PB1 helix-turn-helix motif that contains V273 (V273 helix), and the promoter-binding pocket of the influenza virus RdRp (PDB accession number 5MSG). For clarity, only the right side of the RdRp is shown. (B) Superposed structures of the bat IAV priming loop (PDB accession number 4WSB), influenza B virus priming loop (PDB accession numbers 5MSG, 4WSA, 4WRT, and 5EPI), and influenza C virus priming loop (PDB accession number 5D98). The thickness of the backbone is scaled by the β-factor. Deleted portions of the priming loop are shaded in grays and labeled a to e. (C) Alignment of the PB1 amino acid sequences that constitute the priming loop of the influenza A/WSN/33 (H1N1), influenza B/Michigan/22687/09, and influenza C/JJ/50 virus RdRps. Colors and secondary-structure annotations are described in the legend of Fig. 2B. Deletions in the priming loop are indicated with dotted lines. Labels are based on data shown in panel B. (D) Analysis of steady-state segment 6 viral RNA levels. The graph shows the mean RNA levels, with error bars indicating standard deviations (*n* = 3). The PB1a signal was subtracted as background. (E) SDS-PAGE of recombinant influenza A virus RdRps purified from HEK 293T cells, followed by silver staining or Western blot analysis of PB1 and PA in recombinant RdRps. The bottom two panels show the expression of PB1 and PA RdRp subunits in HEK 293T cells and a tubulin loading control. (F) Schematic of the smFRET promoter-binding assay. Conformations of the influenza virus vRNA promoter before and after binding are shown, as is the distance between the Atto647N (red) and Cy3 (orange) dyes on the two promoter strands. (G) vRNA promoter binding by the IAV RdRp as analyzed by smFRET and fitting with a single Gaussian model. The mean apparent FRET (E*) value of the RNA-only signal is indicated as a dotted line in each graph for reference. (H) Cleavage of a radiolabeled capped 20-nucleotide-long RNA. Alternative cleavage products are indicated with an asterisk.

Research on other RNA virus RdRps has shown that the correct positioning of the template in the active site is dependent on the priming loop (22–24). In the IAV RdRp, the priming loop resides downstream of the active site (Fig. 4A), above the helix-turn-helix containing PB1 V273. To investigate whether the priming loop played a role in priming and realignment, we engineered seven deletions in the influenza A/WSN/33 (H1N1) virus PB1 subunit based on the different conformations of the priming loop in current crystal structures (Fig. 4B) and the sequence conservation of the priming loop among influenza A, B, and C viruses (Fig. 4C). The deleted sections (Δ; sections a to e) are indicated on the crystal structure of the bat influenza A virus RdRp and a PB1 sequence alignment (Fig. 4B and C). In RNP reconstitutions, all mutants were significantly impaired in viral RNA synthesis (Fig. 4D). Due to the interdependence of viral replication and transcription in cell culture (i.e., some amplification of the template vRNA is required to observe mRNA signals above the background), this result was expected for mutants Δ648–651, Δ642–656, and Δ631–662, because they all lack the tip of the priming loop that is critical for the initiation of cRNA synthesis (13). Indeed, none of these mutations was compatible with virus growth. The impaired activities of mutants Δ656–662, Δ631–642, Δ636–642, and Δ631–635 suggest that the middle as well as the N- and C-terminal anchor points of the priming loop play an important role in RdRp activity.

### Purification of priming loop mutants.

The RNP reconstitution assay described above confirmed that the PB1 priming loop is important for viral RNA synthesis, but due to the interdependence of these activities in cell culture, we were not able to observe effects on transcription or replication. To study replication in more detail, the wild-type RdRp, the PB1a mutant, and our seven deletion mutants were expressed in human embryonic kidney (HEK) 293T cells and purified by IgG chromatography. SDS-PAGE and Western blot analyses of the purified proteins showed that eight of the nine recombinant enzymes were able to form heterotrimers (Fig. 4E). The only exception was mutant Δ631–662, for which the PB1 subunit appeared slightly underrepresented in the trimer and the PB1 Western blot signal (Fig. 4E).

To investigate whether the Δ631–662 priming loop deletion affected RdRp-promoter complex formation, we used a single-molecule Förster resonance energy transfer (smFRET)-based binding assay (13, 25) (Fig. 4F). The FRET distribution of the fluorescently labeled RNA in solution resulted in an apparent FRET population with a mean E* value ± standard deviation of 0.76 ± 0.09 (Fig. 4G), whereas the addition of the wild-type RdRp resulted in a shift to a lower apparent FRET value due to a change in the RNA structure upon binding (Fig. 4F). In contrast, incubation with a promoter-binding mutant (“Tetra” mutant) (13) resulted in no shift in the apparent FRET population (Fig. 4G), demonstrating the specificity of the assay. When we next incubated the fluorescently labeled promoter with mutant Δ632–662, we observed a shift in the FRET population similar that of to the wild-type RdRp (Fig. 4G), suggesting that the deletion of the priming loop did not affect promoter binding.

Next, we assessed whether the conformational change that the cap-binding, endonuclease, and 627 domains undergo upon promoter binding (1, 7) was impaired by the priming loop truncations. Since this rearrangement is crucial for cap cleavage, we incubated the priming loop mutants with a 20-nt-long radiolabeled capped RNA. We observed that the activities of the Δ631–662, Δ656–662, Δ631–642, and Δ631–635 mutants were greatly impaired (Fig. 4H and Table 2). In contrast, the activities of mutants Δ648–651, Δ642–656, and Δ636–642 were indistinguishable from that of the wild type (Fig. 4H). Together, data for these controls suggest that mutants Δ648–651, Δ642–656, and Δ636–642 were folded, bound the viral promoter correctly, and were active, while the other four mutants had an unknown impairment that frustrated either cap cleavage or the conformational rearrangement of the RdRp domains.

**TABLE 2:** Overview of PB1 mutant characteristics relative to the wild type^*a*^

Enzyme	RNP reconstitution		Extension on vRNA	Extension on cRNA		RNA cleavage	Trimer formation
	vRNA	mRNA		FL	FR		
Wild type	+++	+++	+++	+++	+++	+++	+++
S269A	+	+	+++	+++	+++	NT	+++
L271A	NT	NT	NT	+++	+++	NT	+++
P272A	NT	NT	NT	−	−	NT	+++
V273A	+	−	+++	+++	++++	NT	+++
Δ648–651	−	−	++++	++++	++++	+++	+++
Δ631–662	−	−	−	−	−	−	+
Δ642–656	−	−	++++	+	++++	+++	+++
Δ656–662	−	−	−	−	−	−	+++
Δ631–642	−	−	+	−	+++	+	+++
Δ636–642	−	−	+++	++	++++	+++	+++
Δ631–635	−	−	+	−	+	+	+++

a ++++, >100% of the value for the wild type; +++, 75 to 100% of the value for the wild type; ++, 50 to 75% of the value for the wild type; +, 25 to 50% of the value for the wild type; −, 0 to 25% of the value for the wild type; NT, not tested.

### The priming loop is important for priming and realignment.

**FIG 5:**
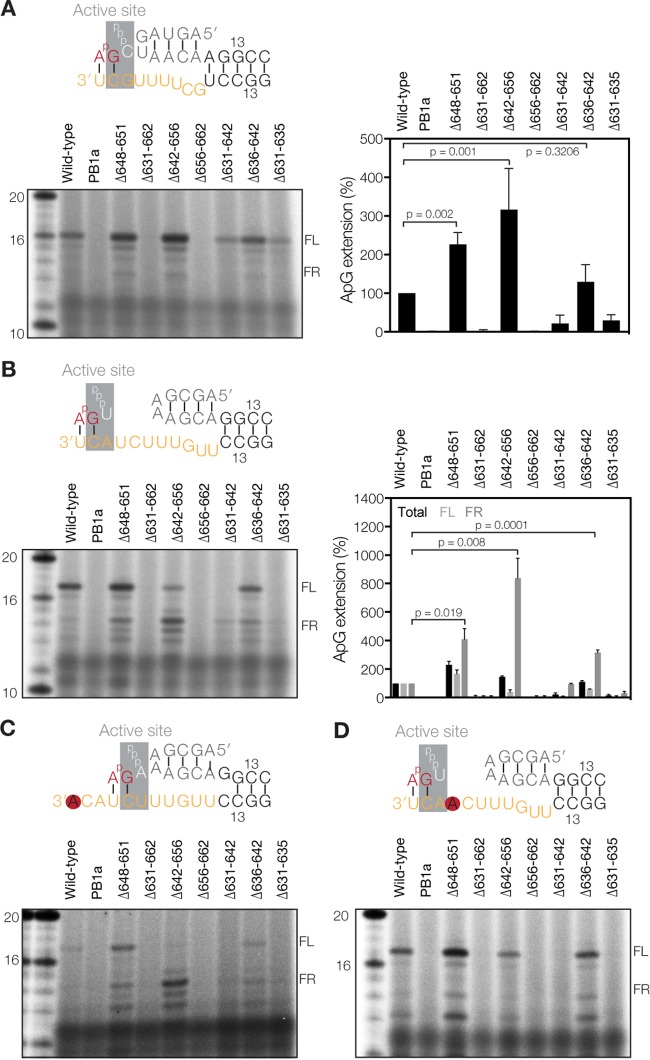
Deletions in the priming loop affect realignment *in vitro*. (A) ApG extension on the vRNA promoter and quantitation of the total vRNA extension activity relative to that of the wild type. (B) ApG extension on a cRNA promoter. The graph shows quantitation of the total ApG extension activity and the production of individual FR or FL bands relative to the wild type. (C) ApG extension on the mutant cRNA promoter 1U→A. (D) ApG extension on the mutant cRNA promoter 4U→A. Graphs show mean activities, with error bars indicating standard deviations (*n* = 3). In panels A and B, *P* values were determined by using an unpaired *t* test.

To investigate the effect of the priming loop deletions on the elongation of the RdRp, we first analyzed the activity of the mutants in the presence of the vRNA promoter. Mutants Δ648–651 and Δ642–656 exhibited higher activity than that of the wild-type enzyme, while the activity of the Δ636–642 mutant was indistinguishable from that of the wild type (Fig. 5A). In all three reactions, no differential change in the product pattern was observed (Fig. 5A). The four remaining priming loop mutants showed greatly impaired activities compared to that of the wild type (Fig. 5A).

We next tested the effect of the mutations on priming and realignment by performing ApG extension assays on the cRNA promoter. We observed that mutants Δ648–651 and Δ642–656 exhibited higher total activity than that of the wild-type enzyme but that this signal contained more incorrectly realigned RNA, as shown by the significantly stronger FR band (Fig. 5B). In the case of mutant Δ636–642, the total activity was composed of a strong increase in FR product synthesis and a reduction in FL product formation. The activity of the remaining four priming loop mutants was again greatly impaired (Fig. 5B), in line with the effect on cap cleavage (Fig. 4H) and ApG extension on the vRNA promoter (Fig. 5A).

To verify that the observed FR product was synthesized due to a failure of the priming and realignment mechanism, we replaced the wild-type cRNA promoter with either the 1U→A promoter to reduce realignment (Fig. 5C) or the 4U→A promoter to prevent internal elongation (Fig. 5D). We found that mutants Δ648–651, Δ642–656, and Δ636–642 were still able to produce the incorrectly realigned RNA on the 1U→A promoter (Fig. 5C). In contrast, on the 4U→A promoter, the Δ642–656 mutant showed a dramatic decrease in the FR band, whereas the Δ648–651 and Δ636–642 mutants still produced some FR signal (Fig. 5D). Interestingly, mutant Δ648–651 was also able to produce an FL product on the 1U→A promoter, while the other mutants and the wild-type polymerase were not, suggesting that this mutant had an increased tolerance for mismatches between the template and the dinucleotide primer (Fig. 5C). Overall, these results are consistent with a model in which the priming loop stimulates realignment during IAV vRNA synthesis and plays a role in suppressing internal elongation, thereby contributing to correct vRNA synthesis.

## DISCUSSION

**FIG 6:**
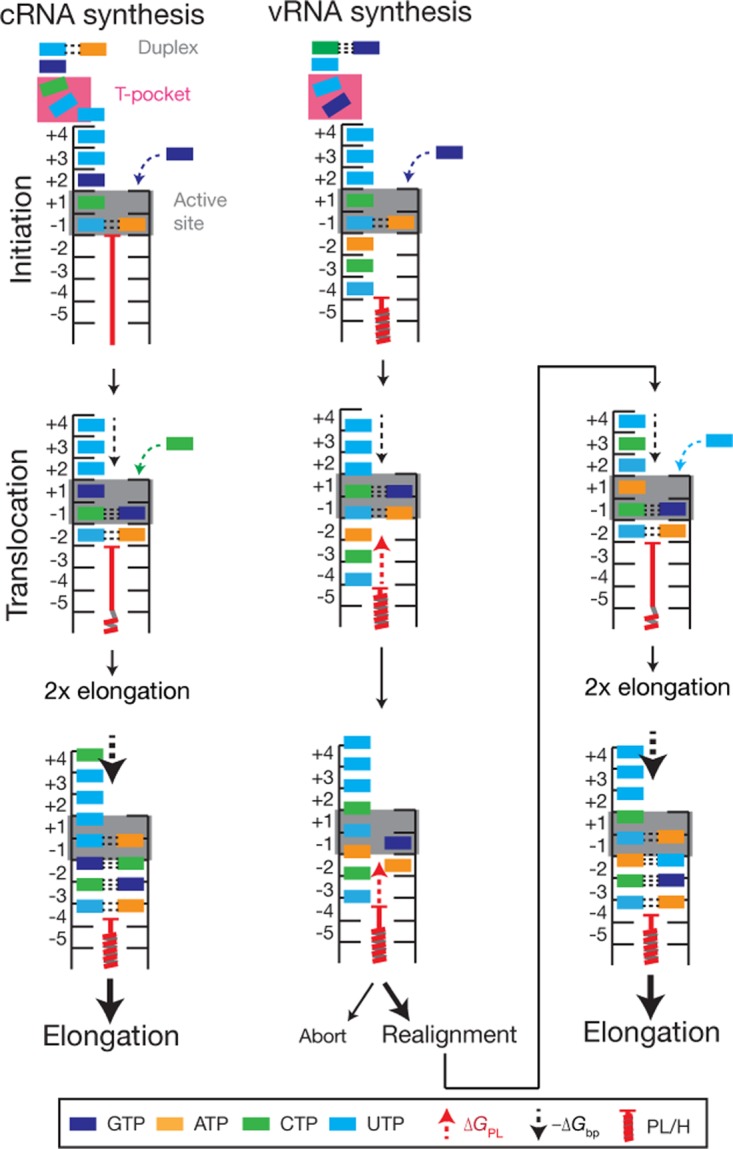
Model of influenza A virus replication initiation. cRNA synthesis is initiated on 3′ 1U and 2C of the vRNA when these residues occupy positions −1 and +1 of the active site. Residues 7U and 8C are stacked in a T-orientation by residues of the PB2 subunit (the “T-pocket”), which likely fixes this end of the vRNA in the RdRp. Indeed, it is likely that the interaction between PB2 and 7U is sequence/structure specific, as a 7U→A mutation was previously shown to abrogate *de novo* initiation (14). To stabilize the initiating ATP, an interaction with the tip of the priming loop is required. Translocation is not hindered by the priming loop after initiation, allowing two elongation steps until a tetramer is formed. A further NTP incorporation event then induces a conformational change in the priming loop, potentially similar to hepatitis C virus (HCV) elongation (26), in order to facilitate progressive elongation. The stability of the template-product duplex (−Δ*G*_bp_) is high enough to overcome a translocation block by the priming loop (Δ*G*_PL_). In contrast, the initiation of a nascent vRNA is catalyzed by using cRNA residues 3′ 4U and 5C as the template when these residues occupy positions −1 and +1 of the active site. Although no structure of the RdRp bound to the complete cRNA promoter is available, it is plausible that G9 will occupy the T-pocket when the cRNA 3′ end enters the template channel, fixing one end of the cRNA in place. Indeed, previous mutational data have shown that like 7U in the vRNA, G9 in the cRNA is essential for *de novo* initiation (14). After dinucleotide formation, the priming loop blocks the translocation of 3′ 1U to position −5, which induces the melting of the template-dinucleotide duplex. The cRNA 3′ terminus can now move back 2 positions until 3′ 1U occupies position −2 to facilitate hybridization with the dinucleotide and allow extension. vRNA extension now becomes effectively similar to cRNA synthesis. The influences of the priming loop/palm subdomain helix (PL/H) structures are drawn as a single red coiled structure for simplicity. The probability of realignment or elongation is indicated by the font size.

The two initiation mechanisms that drive IAV replication are substantially different. *De novo* initiation on a vRNA promoter occurs on the terminus of the template and critically depends on the PB1 priming loop (Fig. 1B and 6) (13). In contrast, *de novo* initiation on the cRNA promoter uses internal residues as the template (13) (Fig. 1B and 6) and requires a realignment step to translocate the nascent vRNA to the terminus of the cRNA promoter before extension can take place. Here we show that a failure to efficiently perform this realignment step generates vRNAs lacking the first 3 nucleotides of the canonical vRNA 5′ terminus, which in turn impairs viral cRNA and mRNA synthesis in cell culture (Fig. 3 and Table 2). In addition, we provide insight into the mechanism controlling this process by demonstrating that the priming loop plays a critical role in the efficiency of the realignment step of the mechanism (Table 2). Based on observations of other RNA virus RdRps, in which the priming loop must undergo a conformational change to allow the product duplex to leave the active site (26, 27), we propose that the priming loop acts as a “spring” that needs to undergo a conformational change to allow efficient elongation of the nascent replication product (Fig. 6). Thus, when the nascent vRNA is short and bound internally on the cRNA, the nascent vRNA cannot induce a conformational change in the priming loop. As a result, the nascent vRNA is destabilized, enabling realignment. However, when the duplex is extended to at least 4 nt, which can occur only when terminal elongation takes place, a conformational change in the priming loop can be induced, allowing the nascent RNA to exit the active site (Fig. 6).

The above-described process relies on the correct positioning of the cRNA terminus in the active site. In a recently reported influenza B virus transcription initiation structure, the vRNA 3′ strand was shown to enter the template entry channel with 6 nt (18) (Fig. 4A), overshooting positions −1 and +1 of the active site by 1 nt. From this position, the template must move back 1 nt to allow terminal *de novo* initiation (Fig. 6). In contrast, to support internal *de novo* initiation, 8 nt must enter the template entry channel (Fig. 6). The IAV RdRp can readily achieve this for the cRNA template, because the cRNA 3′ promoter strand is 1 nt longer than the vRNA 3′ strand, and the promoter duplex is 1 nt shorter (Fig. 1B and 6). Moreover, our data show that reducing the stability of the cRNA promoter duplex does not increase initiation on a cRNA promoter (Fig. 1). Together, these different lines of research suggest that 4U and C5 of the cRNA 3′ promoter strand can be placed at positions −1 and +1 of the active site without duplex unwinding.

After internal *de novo* initiation, the dinucleotide-template duplex melts, and the 3′ terminus of the cRNA template must move back to allow the pppApG dinucleotide to rebind at 1U and 2C at positions −1 and +1 of the active site (Fig. 6). We previously reported that the 3′ end of the cRNA promoter can move freely in and out of the template channel using single-molecule experiments (12). Here we add evidence that the priming loop and palm subdomain residue V273 may stimulate this process (Fig. 2 and 5) by acting as an elongation block (Fig. 6). Importantly, the priming loop does not induce realignment after terminal initiation on a vRNA promoter (Fig. 5), which is consistent with the idea that the stability of the vRNA template-tetramer duplex is sufficient to prevent duplex melting and induce a conformational change in the priming loop (Fig. 6).

In the absence of a structure of the influenza virus RdRp elongation complex, we can only speculate about the mechanism through which V273 affects realignment. Interestingly, in both the apo structure of the influenza C virus RdRp (7) and the influenza B virus RdRp bound to the cRNA 5′ terminus (11), the PB2 cap-binding domain closes the nascent strand exit channel via interactions with the PB1 helix-turn-helix, in which V273 resides, and the PB2 mid-domain (1, 7). If the conformation of the RdRp that has bound the cRNA 5′ terminus is indeed representative of the replicative form of the IAV RdRp, the PB2 cap-binding domain must undergo a conformational change to allow the nascent strand to exit the polymerase. In support of this idea, a recent study showed that a deletion of the cap-binding domain affects vRNA synthesis but not cRNA synthesis (28). It is thus tempting to speculate that V273 may be involved in or positioned through the packing of the PB2 cap-binding domain against the template exit channel and thereby indirectly affecting elongation and realignment. Future studies are needed to address this model in more detail.

In summary, here we have analyzed how the IAV RdRp controls realignment during viral replication, a process that is a critical step in influenza virus RNA synthesis. Our findings offer new insights into influenza virus replication, and they may have implications for the many other RNA viruses that also rely on a priming and realignment mechanism to replicate and/or transcribe their genome (16, 29–31).

## MATERIALS AND METHODS

### Cells and plasmids.

HEK 293T cells were mycoplasma tested and maintained in Dulbecco's modified Eagle's medium (DMEM) (Sigma) supplemented with 10% fetal calf serum (FCS). Plasmids pPolI-NA, pcDNA-NP, pcDNA-PB1, pcDNA-PA, pcDNA-PB2-TAP, and pcDNA-PB1a were described previously (32–34). Also, the promoter-binding mutant (PB1 R238A and R672A and PA K572A and K583A [Tetra mutant]) (13), the PB1 Δ648–651 priming loop mutant (13), the PA D108A endonuclease mutant (35), and the PB1 S269A and V273A palm subdomain mutants were reported previously (20). To construct plasmids expressing additional mutant forms of the PB1 subunit, plasmid pcDNA-PB1 was altered by using site-directed mutagenesis with the primers (Life Technologies) listed in Table 1.

### Sequence alignment and structural modeling.

Amino acid sequences of the PB1 subunits of influenza A/WSN/33 (H1N1), influenza B/Michigan/22687/09, and influenza C/JJ/50 viruses were aligned by using ClustalX (36) and visualized by using ESPript (37). To visualize the influenza B virus RdRp crystal structure (PDB accession number 5MSG), PyMOL 1.3 was used. To model the poliovirus 3D^pol^ elongation complex (PDB accession number 3OL7) into the influenza B virus RdRp crystal structure (PDB accession number 5MSG), we aligned active-site residues 324 to 332 of the poliovirus enzyme with residues 442 to 449 of the influenza virus PB1 protein in PyMOL 1.3.

### Purification of recombinant influenza virus RNA polymerase.

Wild-type and mutant recombinant RdRp preparations were purified by using tap affinity purification (TAP) tags on the C terminus of the PB2 subunit (34). The recombinant polymerases were expressed via transfection of 3 μg of PB1 or mutant PB1, PB2-TAP, and PA into HEK 293T cells using Lipofectamine 2000 (Invitrogen). After 48 h, the cells were lysed, and the recombinant protein was purified by using IgG-Sepharose (GE Healthcare) chromatography and cleavage by tobacco etch virus (TEV) protease (Invitrogen) in cleavage buffer (20 mM HEPES [pH 7.5], 150 mM NaCl, 0.1% NP-40, 10% glycerol, 1× phenylmethylsulfonyl fluoride [PMSF], and 1 mM dithiothreitol [DTT]). The recombinant RdRp preparations were analyzed by 8% SDS-PAGE and silver staining using a SilverXpress kit (Invitrogen). The concentration of the proteins was estimated in gel using a bovine serum albumin (BSA) standard. For Western blotting, polyclonal PB1 (catalog number GTX125923; Genetex), PB2 (catalog number GTX125926; Genetex), and PA (catalog number GTX118991; Genetex) antibodies were used. For activity assays, the RdRp preparations were stored in cleavage buffer at −80°C. RdRp preparations for smFRET experiments were concentrated in a solution containing 50 mM HEPES (pH 7.5), 10% glycerol, 500 mM NaCl, 0.05% *n*-octyl-β-d-thioglucopyranoside (OTG), and 0.5 mM tris(2-carboxyethyl)phosphine (TCEP) before storage at −80°C.

### RNP reconstitution assay.

RNP reconstitutions were performed by transfecting plasmids expressing PB1 or mutant PB1, PA, PB2, and NP together with pPolI-NA into HEK 293T cells by using Lipofectamine 2000 (Invitrogen). Typically, 0.25 μg of each plasmid was used for a 6-well transfection. Total RNA was isolated by using Tri reagent (Sigma) 24 h after transfection and analyzed by using primer extension assays as described previously (13). Briefly, viral RNA species and a 5S rRNA loading control were reverse transcribed by using ^32^P-labeled primers 1280NA, NA160, and 5S100 (Table 1) and SuperScript III (Invitrogen). cDNA synthesis was stopped with 10 μl loading buffer (90% formamide, 10% double-distilled water [ddH_2_O], 10 μM EDTA, xylene cyanol, bromophenol blue) and analyzed by 6% denaturing PAGE (6% 19:1 acrylamide-bisacrylamide, 1× Tris-borate-EDTA [TBE] buffer, 7 M urea). The viral RNA species and the 5S rRNA signal were visualized by using a FLA-500 scanner (Fuji) and analyzed by using AIDA software (RayTek).

### *In vitro* ApG extension assay.

ApG extension assays were performed as described previously (13). Briefly, reactions were performed with 4-μl mixtures that contained 500 μM ApG (Jena Bioscience), 1 mM DTT, 500 μM UTP (unless indicated otherwise), 500 μM CTP, 500 μM ATP (unless indicated otherwise), 0.7 μM vRNA or cRNA promoter (Sigma), 5 mM MgCl_2_, 1 U μl^−1^ RNasin (Promega), 0.05 μM [α-^32^P]GTP (3000 Ci mmol^−1^; Perkin-Elmer), 5% glycerol, 0.05% NP-40, 75 mM NaCl, 10 mM HEPES (pH 7.5), and ∼2 ng RdRp μl^−1^. The reaction mixtures were incubated for 1 h at 30°C, and the reactions were stopped with 4 μl loading buffer. The RNA products were analyzed by 20% denaturing PAGE and visualized by phosphorimaging. *P* values were determined by using an unpaired parametric *t* test.

### Capping of the RNA primer and capped oligonucleotide cleavage assay.

A synthetic 5′-triphosphate-containing 20-nt-long RNA (ppAAUCUAUAAUAGCAUUAUCC; Chemgenes) was capped with a radiolabeled cap-1 structure by using 0.25 μM [α-^32^P]GTP (3,000 Ci mmol^−1^; Perkin-Elmer), 2.5 U/μl 2′-*O*-methyltransferase (NEB), and a vaccinia virus capping kit (NEB), according the manufacturers' instructions. The product was analyzed by 20% denaturing PAGE, excised from the gel, and desalted by using NAP-10 columns (GE Healthcare) that had been equilibrated with RNase-free water. To test the endonuclease activity of the IAV RdRp, we performed reactions with 3-μl mixtures that contained 1 mM DTT, 0.7 μM vRNA promoter (Sigma), 5 mM MgCl_2_, 1 U μl^−1^ RNasin (Promega), 1,500 cpm capped 20-nucleotide-long RNA primer, 5% glycerol, 0.05% NP-40, 75 mM NaCl, 10 mM HEPES (pH 7.5), and ∼2 ng RdRp μl^−1^. The reaction mixtures were incubated for 1 h at 30°C, and the reactions were stopped with 4 μl loading buffer and analyzed by 20% denaturing PAGE. The capped RNA cleavage products were visualized by phosphorimaging.

### *In vitro* dinucleotide synthesis assay.

The pppApG synthesis activity was measured as described previously (13). Briefly, 3-μl reaction mixtures that contained 1 mM DTT, 350 μM adenosine, 5 mM MgCl_2_, 1U μl^−1^ RNasin, 0.05 μM [α-^32^P]GTP (3000 Ci/mmol; Perkin-Elmer), 0.7 μM vRNA or cRNA promoter (Sigma), 5% glycerol, 0.05% NP-40, 75 mM NaCl, 10 mM HEPES (pH 7.5), and ∼2 ng RdRp μl^−1^ were set up. The reaction mixtures were incubated for 18 h at 30°C, inactivated for 2 min at 95°C, and then treated with 1 U calf intestine alkaline phosphatase (Promega) at 37°C for 30 min. The reactions were stopped with 4 μl loading buffer and analyzed by 20% denaturing PAGE.

### Single-molecule Förster resonance energy transfer.

Promoter binding was measured as described previously (12, 13, 25). Briefly, a Cy3 donor dye was placed on 17U of the 3′ promoter strand, and an Atto647N acceptor dye was placed on 6U of the 5′ strand. The RNA oligonucleotides were synthesized by IBA and labeled, purified, and annealed as described previously (25). The excitation of the donor and acceptor fluorophores was measured by using a custom-built confocal microscope with alternating-laser excitation (ALEX) (38, 39). In a typical experiment, ∼100 nM RdRp was preincubated with 1 nM double-labeled promoter RNA in binding buffer (50 mM Tris-HCl [pH 8.0], 5% glycerol, 500 mM NaCl, 10 mM MgCl_2_, 100 μg/ml BSA) for 15 min at 28°C. Samples were diluted 10-fold in binding buffer before the measurements were performed at excitation intensities of 250 μW at 532 nm and 60 μW at 635 nm. The E* values were plotted as one-dimensional distributions and fitted with a single Gaussian model to obtain the mean E* and the standard deviation.
